# Pre-operative methicillin-resistant *Staphylococcus aureus* colonization increases the risk of 90-day medical and 2-year implant-related complications following primary total shoulder arthroplasty: a propensity-matched analysis

**DOI:** 10.1016/j.jsea.2026.100011

**Published:** 2026-03-20

**Authors:** Muhammad Hamza Ilyas, Tarishi Parmar, Zina Smadi, Viraj A. Deshpande, Akin A. Adio, Joseph A. Abboud, Hafiz F. Kassam

**Affiliations:** a Massachusetts General Hospital, Boston, MA, USA; b Division of Shoulder and Elbow Surgery, Rothman Orthopaedic Institute, Philadelphia, PA, USA; c Texas Children's Hospital, Department of Pediatric Orthopedics, Houston, TX, USA; d Sidney Kimmel Medical College, Thomas Jefferson University, Philadelphia, PA, USA; e Perelman School of Medicine, University of Pennsylvania, Philadelphia, PA, USA; f Department of Surgery, Hoag Orthopedics Institute, Irvine, CA, USA

**Keywords:** MRSA, Infection, Total shoulder arthroplasty, Periprosthetic joint infection, Revision, Postoperative complications, Preoperative screening

## Abstract

**Background:**

Pre-operative methicillin-resistant *Staphylococcus aureus* (MRSA) colonization has been associated with increased risk of post-operative infection and medical complications in lower-extremity arthroplasty; however, its impact on outcomes following total shoulder arthroplasty (TSA) remains poorly understood. This study aims to evaluate the association between MRSA colonization and early medical as well as mid-term implant-related complications after primary TSA.

**Methods:**

The TriNetX US Research Network was queried to identify primary TSA patients. Propensity score matching (1:1) was performed using demographic characteristics and relevant comorbidities. Ninety-day medical complications, including readmission, emergency department utilization, venous thromboembolism, pneumonia, urinary tract infection, stroke, sepsis, respiratory failure, and cardiac events, were assessed. Two-year implant-related outcomes included periprosthetic joint infection, aseptic loosening, dislocation, periprosthetic fracture, revision TSA, and all-cause mortality. Risk ratios (RRs) with 95% confidence intervals (CIs) were generated for all comparative analyses.

**Results:**

Overall, 122,665 patients met eligibility criteria. Matching resulted in 2 well-balanced cohorts of 1,781 patients each. At two years, MRSA colonization was associated with significantly higher rates of periprosthetic joint infection (RR, 6.64; 95% CI, 3.53-12.47), aseptic loosening (RR, 1.63; 95% CI, 1.08-2.47), revision surgery (RR 1.75; 95% CI, 1.22-2.52), dislocation (RR, 1.76; 95% CI, 1.08-2.86), and mortality (RR, 1.90; 95% CI, 1.55-2.33). Ninety-day medical complications were also significantly higher among MRSA-colonized patients, with higher rates of readmission, emergency department utilization, venous thromboembolism, pulmonary embolism, pneumonia, urinary tract infection, stroke, sepsis, and respiratory failure (all *P* < .01). Cardiac events occurred at similar rates between cohorts (*P* = .390).

**Conclusion:**

Pre-operative MRSA colonization was associated with substantially higher risks of both 90-day medical and 2-year implant-related complications following TSA. These findings highlight the importance of routine MRSA screening, consideration of decolonization protocols, and heightened perioperative vigilance in this high-risk population.

Total shoulder arthroplasty (TSA) is one of the most common and rapidly growing orthopedic procedures worldwide, with an estimated annual growth rate of 11.65% in the United States.^13^ By 2026, the demand for primary TSA is projected to reach over 10 million procedures annually.^8^ Despite these advances, periprosthetic joint infection (PJI) remains one of the most devastating complications following TSA and represents the third leading cause of revision surgery—occurring in up to 3% of reverse TSAs and 0.5%-1% of anatomic TSAs.^12^^,^^34^ PJIs are associated with poorer functional outcomes, increased morbidity, and higher mortality, underscoring the need for continued efforts in risk stratification and prevention.^9^

Pre-operative bacterial colonization has been identified as an important modifiable risk factor for PJI and subsequent revisions.^30^^,^^33^ Culture-positive PJIs have been reported in approximately 64.5% of hip and knee arthroplasty infections, with causative organisms including Gram-positive bacteria (50.4%), Gram-negative bacteria (23.1%), and fungi (0.8%).^10^ Among these, methicillin resistance was identified in 64.3% of *Staphylococcus aureus* isolates and 89.5% of coagulase-negative staphylococci. Methicillin-resistant *S. aureus* (MRSA) has emerged as an increasingly prevalent pathogen, mirroring the global trend of rising antibiotic resistance, by which the incidence of MRSA-related PJIs continues to increase, whereas coagulase-negative staphylococci-related infections are declining.^14^ MRSA accounts for approximately 5.1% of shoulder PJIs and up to 21% of hip and knee PJIs, with MRSA colonization leading to 4 times greater risk of PJI in total joint arthroplasty (TJA).^6^^,^^20^

Furthermore, pre-operative MRSA colonization has been linked to longer hospital stays, higher readmission rates, and increased rates of both septic and aseptic revisions among patients undergoing TJA. In total knee arthroplasty specifically, MRSA colonization has also been associated with a greater risk of wound-related complications.^3^ As a result, pre-operative MRSA screening has become the standard of care in TJA.^38^

However, evidence regarding the impact of pre-operative MRSA colonization on TSA outcomes remains limited. Therefore, this study aims to investigate 90-day medical and 2-year implant related complications in patients with pre-operative MRSA colonization undergoing TSA. By understanding such effects, we aim to improve pre-operative screening for patients undergoing TSA and therefore enhance perioperative management and outcomes for MRSA carriers. We hypothesized that patients colonized with MRSA would demonstrate significantly higher rates of both early medical and midterm implant-related complications following TSA.

## Methods

### Data source

Data for this study were obtained from the TriNetX US Research Network, a federated database comprising electronic health records from more than 70 health care organizations and over 130 million patients nationwide. The network provides access to longitudinal clinical data relevant to musculoskeletal conditions and surgical procedures, including TSA. All data were deidentified in accordance with the Health Insurance Portability and Accountability Act of 1996 Privacy Rule (§164.514[a]), with deidentification procedures verified through a formal determination by a qualified expert (§164.514[b][1]). This investigation was conducted as a retrospective analysis of deidentified data and was therefore exempt from informed consent and institutional review board approval.

### Cohort selection

The TriNetX US Research Network was queried to identify patients who underwent primary TSA between January 1, 2005, and January 1, 2023. Eligible patients were required to be 18 years of age or older and to have undergone a primary TSA, identified using Current Procedural Terminology (CPT) code 23472. Patients with proximal humeral fractures, malignant neoplasms of the upper extremity, pathological fractures, or less than 2 years of follow-up were excluded. Patients were stratified according to pre-operative colonization with MRSA. MRSA-colonized individuals were identified through documentation of a carrier state or a positive MRSA culture within 1 year preceding the index TSA, based on International Classification of Diseases, Tenth Revision (ICD-10) codes. Patients without any such documentation comprised the noncolonized comparator cohort.

### Propensity score matching

Propensity score matching was performed to reduce selection bias and improve comparability between MRSA-colonized and noncolonized cohorts. Matching was conducted using a 1:1 nearest-neighbor algorithm without replacement, with a caliper width of 0.1, and was based on demographic variables and relevant comorbidities, including diabetes mellitus, hypertension, hyperlipidemia, chronic kidney disease, heart failure, liver disease, osteoporosis, tobacco use, and body mass index (BMI).

### Outcome measures

Post-operative outcomes were assessed at 90 days and 2 years following TSA. Outcomes were identified using ICD-10 diagnostic codes and CPT procedure codes. Ninety-day outcomes included emergency department (ED) utilization, readmissions, and medical complications, including venous thromboembolism (deep vein thrombosis and pulmonary embolism), pneumonia, urinary tract infection, stroke, sepsis, respiratory failure, and cardiac events, including acute myocardial infarction and cardiac arrest. Two-year outcomes included PJI, aseptic loosening, dislocation, periprosthetic fracture, revision TSA, and all-cause mortality.

### Statistical analysis

Descriptive statistics were used to summarize demographic and clinical variables. Categorical variables were compared using risk ratios (RRs) with 95% confidence intervals (CIs), and continuous variables were compared using *t*-tests as appropriate. Statistical significance was defined as *P* < .05. All analyses were performed within the TriNetX real-time analytics platform.

## Results

### Baseline characteristics

Before matching, the MRSA-colonized cohort included 1,781 patients, whereas the noncolonized cohort included 120,884 patients undergoing primary TSA. Significant baseline differences were observed across numerous demographic and clinical characteristics. Patients with MRSA colonization were more likely to have diabetes mellitus (39.9% vs. 19.2%, *P* < .001), hypertension (76.4% vs. 52.4%, *P* < .001), hyperlipidemia (53.8% vs. 35.7%, *P* < .001), heart failure (26.9% vs. 7.8%, *P* < .001), chronic kidney disease (27.5% vs. 9.6%, *P* < .001), liver disease (17.0% vs. 7.0%, *P* < .001), osteoporosis (17.2% vs. 9.0%, *P* < .001), tobacco use (7.4% vs. 2.8%, *P* < .001), and alcohol use (2.0% vs. 0.9%, *P* < .001). Differences were also present in age (67.8 ± 11.7 vs. 68.4 ± 11.1 years, *P* = .028), sex distribution (female: 55.2% vs. 52.5%, *P* = .022), racial categories, including higher proportions of Black or African American patients (9.2% vs. 7.6%, *P* = .012) and lower proportions of Hispanic/Latino patients (1.9% vs. 3.0%, *P* = .005), and BMI (31.8 ± 8.1 vs. 30.7 ± 6.7 kg/m^2^, *P* < .001).

After 1:1 propensity score matching, 1,781 patients remained in each cohort. Matching eliminated all statistically significant imbalances, with no remaining differences across demographic variables, comorbidities, or BMI (all *P* > .05), indicating adequate covariate balance (Table I).

**Table I:** Baseline characteristics before and after matching between MRSA-colonized and noncolonized shoulder arthroplasty patients.

Variable	Before matching		*P* value	After matching		*P* value
	MRSA colonized cohort (%) or mean ± SD	Non-MRSA colonized cohort (%) or mean ± SD		MRSA colonized cohort (%) or mean ± SD	Non-MRSA colonized cohort (%) or mean ± SD	
Age at index	67.8 ± 11.7	68.4 ± 11.1	.028	67.8 ± 11.7	68.3 ± 11.5	.234
Female	55.2	52.5	.022	55.2	55.6	.813
Male	44.8	47.5	.025	44.8	44.4	.787
Black or African American	9.2	7.6	.012	9.2	9.1	.907
White	85.8	85.1	.431	85.8	87.0	.305
American Indian or Alaska Native	0.6	0.4	.181	0.6	0.6	1.000
Asian	0.6	0.8	.237	0.6	0.6	1.000
Other race	1.0	2.3	<.001	1.0	1.0	1.000
Unknown race	2.9	3.6	.153	2.9	2.1	.135
Not Hispanic or Latino	76.7	74.9	.091	76.7	77.7	.472
Hispanic or Latino	1.9	3.0	.005	1.9	1.5	.298
Unknown ethnicity	21.4	22.0	.535	21.4	20.8	.681
Diabetes mellitus	39.9	19.2	<.001	39.9	41.3	.394
Essential hypertension	76.4	52.4	<.001	76.4	77.6	.381
Hyperlipidemia	53.8	35.7	<.001	53.8	55.5	.313
Heart failure	26.9	7.8	<.001	26.9	26.8	.970
Chronic kidney disease	27.5	9.6	<.001	27.5	27.9	.764
Liver disease	17.0	7.0	<.001	17.0	15.9	.416
Osteoporosis age related	17.2	9.0	<.001	17.2	17.3	.929
Tobacco use	7.4	2.8	<.001	7.4	6.6	.358
Alcohol use unspecified	2.0	0.9	<.001	2.0	2.0	1.000
BMI	31.8 ± 8.1	30.7 ± 6.7	<.001	31.8 ± 8.1	31.8 ± 7.3	.991

*SD*, standardized difference; *BMI*, body mass index; *MRSA*, methicillin-resistant *Staphylococcus aureus*.

All values are expressed as mean ± SD or percentage unless otherwise indicated. After matching, all standardized differences were less than 0.1, confirming adequate covariate balance.

### Primary outcomes

At two years, MRSA colonization was associated with substantially higher implant-related complications following TSA. The rate of PJI was markedly elevated among colonized patients (4.1% vs. 0.6%; RR, 6.64; 95% CI, 3.53-12.47; *P* < .001). Aseptic loosening (3.2% vs. 2.0%; RR, 1.63; 95% CI, 1.08-2.47; *P* = .020) and revision surgery (4.3% vs. 2.5%; RR, 1.75; 95% CI, 1.22-2.52; *P* = .002) were likewise observed at significantly higher rates in the MRSA cohort. Dislocation occurred more frequently among colonized patients (2.5% vs. 1.4%; RR, 1.76; 95% CI, 1.08-2.86; *P* = .021). Although the rate of periprosthetic fracture was higher in MRSA-colonized patients (1.3% vs. 0.7%; RR, 1.85; 95% CI, 0.94-3.61), this difference did not reach statistical significance (*P* = .069). All-cause mortality was nearly doubled at two years among colonized patients (13.6% vs. 7.2%; RR, 1.90; 95% CI, 1.55-2.33; *P* < .001) (Table II).

**Table II:** Primary post-operative outcomes in patients with pre-operative MRSA colonization compared with noncolonized patients, with associated relative risks and 95% confidence intervals.

Outcome	MRSA colonized cohort	Non-MRSA colonized cohort	RR [95% CI]	*P* value
PJI	4.10%	0.60%	6.636 [3.533, 12.467]	**<.001**
Aseptic loosening	3.20%	2.00%	1.629 [1.075, 2.468]	**.020**
Revision	4.30%	2.50%	1.750 [1.215, 2.520]	**.002**
Dislocation	2.50%	1.40%	1.760 [1.082, 2.863]	**.021**
Periprosthetic fracture	1.30%	0.70%	1.846 [0.943, 3.614]	.069
Mortality	13.60%	7.20%	1.898 [1.549, 2.327]	**<.001**

*CI*, confidence interval; *RR*, risk ratio; *PJI*, periprosthetic joint infection; *MRSA*, methicillin-resistant *Staphylococcus aureus*.

Bold values indicate *P* < .05.

### Secondary outcomes

Within 90 days following TSA, MRSA-colonized patients experienced significantly higher rates of early medical complications compared with noncolonized counterparts. Readmission was more frequent in the MRSA cohort (4.5% vs. 1.5%; RR, 3.12; 95% CI, 2.01-4.82; *P* < .001), and ED utilization was nearly doubled (25.5% vs. 13.3%; RR, 1.93; 95% CI, 1.67-2.22; *P* < .001). Elevated risks were also observed for deep vein thrombosis (4.5% vs. 1.6%; RR, 2.86; 95% CI, 1.87-4.37; *P* < .001), pulmonary embolism (2.4% vs. 1.2%; RR, 1.96; 95% CI, 1.17-3.25; *P* = .009), pneumonia (8.4% vs. 3.9%; RR, 2.17; 95% CI, 1.65-2.87; *P* < .001), urinary tract infection (8.2% vs. 4.7%; RR, 1.74; 95% CI, 1.34-2.26; *P* < .001), stroke (3.3% vs. 1.9%; RR, 1.79; 95% CI, 1.17-2.72; *P* = .006), sepsis (10.3% vs. 3.0%; RR, 3.39; 95% CI, 2.52-4.56; *P* < .001), and respiratory failure (10.4% vs. 4.5%; RR, 2.31; 95% CI, 1.79-2.98; *P* < .001). No significant difference was observed in cardiac events (3.4% vs. 2.9%; RR, 1.17; 95% CI, 0.82-1.69; *P* = .390) (Table III).

**Table III:** Secondary post-operative outcomes in patients with pre-operative MRSA colonization compared with noncolonized patients, with associated relative risks and 95% confidence intervals.

Outcomes	MRSA colonized cohort	Non-MRSA colonized cohort	RR [95% CI]	*P* value
Readmission	4.50%	1.50%	3.115 [2.013, 4.822]	**<.001**
ED utilization	25.50%	13.30%	1.928 [1.671, 2.224]	**<.001**
DVT	4.50%	1.60%	2.857 [1.867, 4.372]	**<.001**
Pulmonary embolism	2.40%	1.20%	1.955 [1.174, 3.253]	**.009**
Pneumonia	8.40%	3.90%	2.174 [1.647, 2.869]	**<.001**
UTI	8.20%	4.70%	1.738 [1.340, 2.255]	**<.001**
Stroke	3.30%	1.90%	1.788 [1.174, 2.724]	**.006**
Cardiac events	3.40%	2.90%	1.173 [0.815, 1.688]	.390
Sepsis	10.30%	3.00%	3.389 [2.520, 4.558]	**<.001**
Respiratory failure	10.40%	4.50%	2.313 [1.794, 2.981]	**<.001**

*ED*, emegency department; *DVT*, deep vein thrombosis; *UTI*, urinary tract infection; *CI*, confidence interval; *RR*, risk ratio; *PJI*, periprosthetic joint infection; *MRSA*, methicillin-resistant *Staphylococcus aureus*.

Bold values indicate *P* < .05.

## Discussion

In this large multicenter TriNetX analysis of patients undergoing primary TSA, pre-operative MRSA colonization was associated with significantly higher risks of both 90-day medical and 2-year implant-related complications compared to matched noncolonized controls. The most notable increases were observed in PJI, aseptic loosening, revision surgery, dislocation, and mortality at two years, as well as early systemic complications including readmission, ED utilization, venous thromboembolism, pulmonary embolism, pneumonia, urinary tract infection, stroke, sepsis, and respiratory failure within 90 days. These findings collectively indicate that MRSA colonization represents a potent marker of heightened post-operative morbidity and mortality following shoulder arthroplasty.

Our finding of a sixfold increased risk of PJI among MRSA-colonized patients aligns with existing evidence identifying nasal or skin MRSA carriage as one of the strongest predictors of post-operative infection following joint replacement.^15^^,^^23^ *S. aureus* is among the most frequently isolated pathogens in PJIs after rotator cuff repair, and Nelson et al reported it as the causative organism in 14.8% of all PJIs following shoulder arthroplasty.^5^^,^^17^^,^^24^^,^^34^ Studies in hip and knee arthroplasty have similarly shown that pre-operative MRSA colonization confers persistently elevated infection risk, even after adjusting for comorbidities and antibiotic prophylaxis.^15^^,^^23^ Furthermore, MRSA infections are generally more frequent and severe than those caused by methicillin-sensitive strains (MSSA), with risks further amplified in patients with multiple comorbidities undergoing elective orthopedic procedures.^16^^,^^19^^,^^25^^,^^28^ These findings emphasize the importance of routine pre-operative MRSA screening and decolonization using intranasal mupirocin and chlorhexidine washes, which have been shown to significantly reduce Surgical Site Infection rates in arthroplasty populations.^35^

The elevated risk of aseptic loosening and revision procedures in colonized patients may reflect both direct sequelae of infection and chronic inflammatory responses to low-grade bacterial biofilm. These findings are consistent with existing literature. Kapur et al^16^ reported a 12.5% incidence of revision surgery following elective knee and hip arthroplasty due to MRSA-related PJI. Ashkenazi et al^4^ similarly demonstrated higher rates of aseptic loosening among patients undergoing TJA. Clinically, this supports the need for more aggressive intraoperative antisepsis and vigilant post-operative surveillance in MRSA-colonized patients. In addition, the nearly twofold increase in dislocation risk observed in our cohort may be attributable to the immunocompromised status associated with MRSA infection and infection-related soft-tissue compromise, as well as capsular distention and inflammatory destruction, all of which can diminish periarticular support and impair the inherent stability of the prosthesis.^1^^,^^26^^,^^32^

Increased 90-day readmission and ED utilization among MRSA carriers likely reflect post-operative complications related to infection, respiratory issues, and thromboembolic events, which were found to be significantly higher in our MRSA cohort.^21^^,^^38^ Prior evidence has demonstrated that MSSA and MSSA collectively account for approximately 13%-15% of infection-related readmissions following total hip and total knee arthroplasty. Furthermore, MRSA-associated readmissions were shown to independently prolong hospital length of stay by a mean of 3.05 days, underscoring the substantial clinical and resource burden attributable to these infections.^38^ Anderson et al^2^ further reported that post-operative MRSA infection leads to an average of 23 additional hospital days and independently predicts readmission within 90 days. The heightened rates of deep vein thrombosis and pulmonary embolism may be partly driven by the systemic inflammatory milieu associated with infection, which promotes a hypercoagulable state.^22^ These findings support early mobilization and consideration of pharmacologic thromboprophylaxis in MRSA-colonized arthroplasty patients, even when standard venous thromboembolism risk appears low. Our results indicated a significantly higher risk of stroke in the MRSA cohort; however, evidence in the literature remains mixed. Studies collectively suggest that while MRSA infection is associated with higher mortality and systemic complications, it does not independently increase stroke incidence.^11^^,^^14^^,^^31^ Further research is thus warranted to clarify whether MRSA contributes to the cerebrovascular risk in the post-operative setting.

The increased incidences of pneumonia, UTI, and sepsis highlight the systemic vulnerability of MRSA-colonized patients. MRSA carriage has been associated with dysbiosis of the skin and mucosal microbiota, impaired innate immunity, and increased susceptibility to nosocomial infections.^18^^,^^36^ MRSA is a leading cause of both community-acquired and hospital-acquired pneumonia, with hospital-acquired and ventilator-associated forms carrying particularly poor prognoses. In severe cases, it can produce fulminant necrotizing pneumonia, rapidly progressing to sepsis and septic shock.^37^ Furthermore, MRSA exhibits remarkable adaptability across different host environments, enhancing its capacity for systemic invasion. For example, human urine has been shown to modify MRSA virulence and gene expression, promoting persistence and pathogenicity in urinary tract infections.^27^ Importantly, MRSA carriage has been shown to confer a markedly elevated risk of invasive infection, with actively screened carriers demonstrating nearly a 20-fold higher likelihood of developing MRSA bacteremia compared with noncolonized patients, reinforcing its role as a key driver of systemic infectious complications rather than a benign colonizing state.^20^

Collectively, these findings suggest that MRSA colonization reflects not only localized bacterial presence but also an underlying predisposition to severe systemic infection. Furthermore, mortality at two years was nearly doubled among MRSA carriers, consistent with literature demonstrating that MRSA infection and colonization are independently associated with increased all-cause mortality following orthopedic procedures.^7^^,^^29^ This underscores the long-term impact of pre-operative colonization, which may contribute to both infection-related deaths and systemic complications.

Our findings must be interpreted in the context of several important limitations. This study is retrospective in nature, with data derived from electronic health records independent of investigator control, which introduces inherent risks of selection bias, residual confounding, and misclassification. Although propensity score matching was used to mitigate baseline differences between MRSA-colonized and noncolonized cohorts, unmeasured confounders cannot be fully eliminated. MRSA status was identified by the presence or absence of the ICD-10 Clinical Modification code for MRSA colonization (Z22.322), and the platform does not indicate whether universal pre-operative screening was performed. Consequently, patients without documented MRSA status may include unscreened individuals who were truly colonized, introducing potential selection bias and exposure misclassification. Readmissions and ED encounters were ascertained using CPT codes, which do not consistently capture the primary indication for each encounter. As a result, we were unable to distinguish events directly related to infectious complications from those attributable to unrelated medical or surgical conditions. The database does not reliably capture whether patients underwent pre-operative MRSA decolonization with intranasal mupirocin and chlorhexidine washes, thereby limiting the assessment of the prevalence or effect of decolonization within this cohort. Finally, functional outcomes, including patient-reported outcome measures, Minimal Clinically Important Difference, and patient acceptable symptom state, could not be assessed using this platform. Despite these limitations, the study's strengths include a large, multicenter cohort, robust propensity matching, and evaluation of both early medical and midterm implant-related outcomes with extended follow-up. Future studies incorporating granular microbiologic data, perioperative protocols, and patient-reported outcomes are warranted to further refine risk stratification and management strategies for MRSA-colonized patients undergoing TSA.

## Conclusion

In conclusion, MRSA colonization before shoulder arthroplasty significantly increases both early and late complications. From a clinical perspective, routine MRSA screening and decolonization protocols, judicious use of vancomycin prophylaxis, optimized perioperative skin antisepsis, and close post-operative monitoring may help mitigate these risks. Future studies should investigate whether targeted decolonization and antibiotic stewardship protocols can reduce revision and mortality rates among this high-risk population.

## Disclaimers

Funding: No funding was disclosed by the authors.

Conflicts of interest: Joseph Abboud would like to disclose: Royalties from a company or supplier; disclosures; Osteocentric Technologies, Enovis, Zimmer-Biomet, Stryker, Globus Medical, Inc. Stocks in: Shoulder Jam, Aevumed, Oberd, OTS Medical, Orthobullets, Atreon, Restore 3D. Research support from a company or supplier as a PI; disclosures; Enovis, Arthrex. Royalties, financial or material support from publishers; disclosures; Wolters Kluwer, Slack Orthopaedics, Elsevier. Board member/committee appointments for a society; Disclosures; American Shoulder and Elbow Society, Mid Atlantic Shoulder and Elbow Society, Shoulder 360, Pacira. Hafiz Kassam would like to disclose: Consultant for Zimmer Biomet and Smith & Nephew. Any additional authors, their immediate families, and any research foundations with which they are affiliated have not received any financial payments or other benefits from any commercial entity related to the subject of this article.
